# Searching for female reproductive aging and longevity biomarkers

**DOI:** 10.18632/aging.203206

**Published:** 2021-06-22

**Authors:** Svetlana Yureneva, Viktoriya Averkova, Denis Silachev, Andrey Donnikov, Alla Gavisova, Vladimir Serov, Gennady Sukhikh

**Affiliations:** 1 National Medical Research Center for Obstetrics, Gynecology and Perinatology Named after Academician V.I. Kulakov, Moscow 117997, Russia; 2 A.N. Belozersky Research Institute of Physico-Chemical Biology, Moscow State University, Moscow 119992, Russia

**Keywords:** female reproductive aging, biomarkers of reproductive aging, ovarian reserve, biological age

## Abstract

Female reproductive aging is, in a way, a biological phenomenon that develops along canonical molecular pathways; however, it has particular features. Recent studies revealed complexity of the interconnections between reproductive aging and aging of other systems, and even suggested a cause-effect uncertainty between them. It was also shown that reproductive aging can impact aging processes in an organism at the level of cells, tissues, organs, and systems. Women at the end of their reproductive lives are characterized by the accelerated incidence of age-related diseases. Timing of the onset of menarche and menopause and variability in the duration of reproductive life carry a latent social risk: not having enough information about the reproductive potential, women keep on postponing childbirth. Identification and use of the most accurate and sensitive aging biomarkers enable the prediction of menopause timing and quantification of the true biological and reproductive ages of an organism. We discuss current views on reproductive aging and peculiarities of using available biomarkers of aging. We also consider latest advances in the search for potential genetic markers of reproductive aging. Finally, we posit the importance of determining the female biological age and highlight potential research directions in this area.

## INTRODUCTION

It’s a fact of life that every human moves down one and only path, which is aging. This path, as scientists suggest, starts in the embryonic development period — the so-called “zero point” — when both life and aging of an organism begin simultaneously [1]. Research in the area of aging processes, which dates back to ancient times, has not yet led us to a consensus on the definition of this phenomenon, nor has it definitively identified unique biomarkers of such processes [2, 3]. Such uncertainty may be due to the fact that, when studying the complex bi-directional relationship between the processes of aging and disease (which often employ the same mechanisms of loss of cellular stability or ability to recover [4], scientists have to constantly make attempts to split off these biological phenomena or, conversely, to prove they are identical [5].

Currently, the concept implying that aging is not a disease prevails in the academic community. However, we believe that despite the existing conceptual differences, these processes can be neither called parallel nor completely isolated from each other. Having no specific symptoms, unlike disease, aging still has its own, inherent pathological features [6, 7]. Moreover, aging has long been recognized as a risk factor for many diseases [8]. On top of that, aging is associated with accelerated accumulation of medical conditions. If we imagine these processes as straight lines on a plane, then, just as per Euclid's fifth postulate, with the time the person lives these lines will be converging and—sooner or later—will intersect, which will mark the death of the organism. In this concept, every person would have a unique time of intersection and converging trajectories. However, every single person will inevitably reach the ultimate destination of the path—the end of existence.

Interestingly, the attempts of researchers to delve into the deep insights of aging face numerous challenges—particularly because this condition is not regarded as disease. Natural, inevitable, and, most importantly, physiological, this process also does not imply any “treatment.” But, as we all know, “everyone wants to live long yet no one wants to be old.” With greater age comes a heavier burden of conditions and, subsequently, lower quality of life. Potential strategies for “anti-aging” and searching for corresponding biomarkers are becoming increasingly relevant for the aging world population.

To work out these strategies, scientists are trying to simulate the processes that occur in the body with age. Pregnancy seems to be an interesting case to consider (see for review [9]. With underlying temporary medical conditions that are largely similar to aging-associated medical conditions (e.g., hypercoagulability, impaired glucose tolerance, and insulin resistance) as well as molecular processes at the cellular level (oxidative stress, inflammation), pregnancy has unique potential to be a platform for studying the process of aging. At the same time, a useful set of signaling pathways activated by pregnancy [10] and reversibility of pathological phenomena associated with it are of great interest in context of development of “anti-aging” tools. The endogenous nature of potential targeted agents, which, if successfully developed, can be used in pathology of aging, is of great value and makes pregnancy a standout among other models considered earlier [11].

Such studies add to our knowledge of the complex interrelation between aging processes (and hence accumulation of diseases) and hormonal changes in the body. There is much already known about the role of female sex hormone deficiency in development of aging-associated diseases [12]. Reproductive aging, as a cause of a decrease in the level of sex hormones, in this sense, is a unique phenomenon indeed. Developing seemingly along to the canonical biological pathways, this process interrelates with disease in a more complex manner than somatic aging while having a faster progression rate, which has been confirmed by numerous studies.

In particular, recent analysis of mutations in the genomes of various types of cancer showed that the mutation accumulation rate in ovarian and uterine tumor cells is 20 % higher than that in tumors of other tissues, which, after extrapolation suggests a 20 % increase in the reproductive aging rate [13]. The decline in ovarian non-growing follicle number begins in the prenatal period and constantly accelerates thereafter [14] before it reaches the critical threshold of about 1,000 follicles. After this, the menopause ensues—usually occurring long before the death of a woman.

Desynchronization of the reproductive and somatic aging rates causes a woman's post-reproductive lifespan to be about 30 years of her total life expectancy [15, 16]. Since sex hormones produced by the ovarian follicles are directly involved in the processes maintaining the internal homeostasis of the organism, loss thereof leads to many health preservation mechanisms getting out of balance. The latter is followed by changes in the fundamental aging processes at the cellular, tissue, organ, and system levels [17, 18]. Natural menopause, which is an example of naturally developing isolated aging, is intended to terminate the reproductive function rather than end the existence of an individual. Nevertheless, menopause is the point of no return after which a woman starts moving fast down the path of accumulation of diseases. Even more dramatic consequences are associated with premature menopause, a condition that is thought to share with multimorbidity not only loss of sex hormones but also other more complex mechanisms [19].

## Social and economic significance of the search for markers of reproductive aging

Biomarkers are condition indicators that reflect whether an organism or a cell has such condition [20]. The concept of using biomarkers in clinical practice comes down to several aspects: from diagnosing the development probability of a disease or condition to forecasting response to treatment. The search for biomarkers of aging is necessary to assess the progression of aging as well as to evaluate the effect of any drug that influences aging, including anti-aging treatments [21].

It is important to note that the American Federation for Aging Research (AFAR) proposed in 2013 that all biomarkers of aging should meet the criterion of being able to predict the rate of aging [22]. But with regard to reproductive aging, this criterion also acquires special social value, since modern women are increasingly delaying childbearing to a later age. In Russia, the average age of first-time mothers increased to 28.5 years through 2015–2017; for comparison, it amounted to 20.9 years in 1995–1999 [23]. Such a trend, as is commonly known, can be found in all developed countries of America and Europe. Subfertility is one of the earliest clinical signs in the cascade of events associated with reproductive aging. Despite the fact that some studies revealed that optimal and stable probability of monthly conception and giving birth to a live child is there until the age of 31 [24], the age when this probability begins to decrease is even more variable than the age at menopause [25]. The above-mentioned fact clearly suggests that women should be aware of their reproductive potential and plan their families considering the real reproductive age rather than the chronological age.

**Figure 1:**
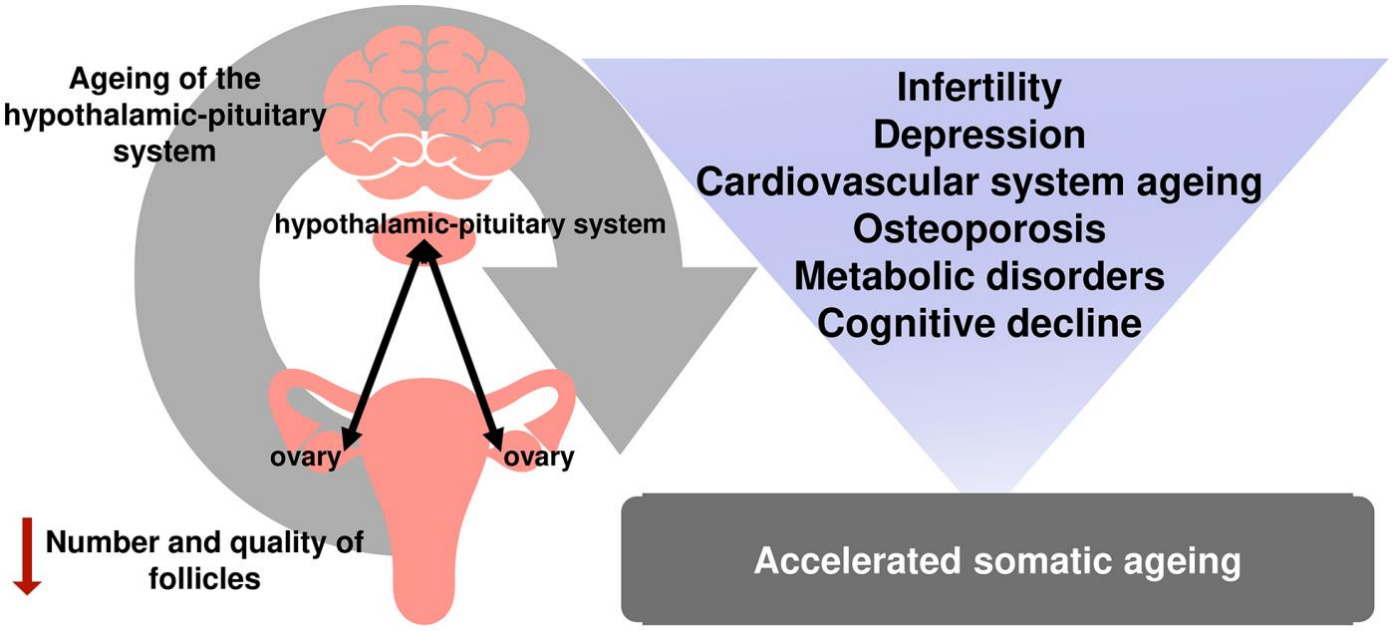
The relationship between reproductive and somatic aging.

At the same time, infertility is just the tip of the iceberg of reproductive aging. Numerous studies have demonstrated that menopause is associated with a higher risk of cardiovascular and other diseases [26, 27]. Menopause is included in the Framingham Risk Score for Women, developed based on the data of an extensive 12-year epidemiological study conducted in the United States [28]. Osteoporosis is yet another known negative health effect caused by estrogen deprivation during reproductive aging in females [29] (Figure 1).

Not only the onset of menopause itself but the time of the onset can significantly contribute to the development of medical conditions in women. The cardiovascular risk is known to increase as the age of menopause onset comes down [19, 30, 31]. Premature ovarian insufficiency (premature menopause) has been recognized as the second most important disease causing an increased risk of osteoporosis [32]. Cognitive decline [33], sexual disorders [34], depressive disorders [35], metabolic disorders, and overall deterioration in quality of life are particularly associated with early-onset and premature menopause [36].

The reproductive lifespan of a woman depends on presence of multiple somatic diseases and all possible causes of death [37, 38]. Duration of the estrogenic effect on the body is most likely to be the key aspect in this regard. Data from The Women's Health Initiative suggest that longer reproductive lifespan is significantly associated with increased overall longevity [37]. The onset of menarche, one of the milestones in the reproductive life, is also a crucial factor in a woman's reproductive lifespan. However, age at menarche affects the risk of progression of somatic diseases in different ways. Early age at menarche (<12 years) has been shown to be associated with the risk of cardiovascular and metabolic disorders [39, 40]. Every 2 years of delay in age at menarche is associated with a 10 % decrease in the risk of breast cancer [41]. However, late age at menarche is associated with an increased risk of developing osteoporosis [42].

The reproductive lifespan varies a lot. The time of the menarche onset depends on hereditary factors, ethnicity, girl's characteristics at birth, factors in play at early infancy, as well as lifestyle and environmental factors [43]. The time of menopause onset certainly depends on the chronological age, but the variety of factors present that influence this event can actually break this link [44]. Moreover, scientists point out the discrepancies in the initial supply of follicles at the time of birth of a girl as one of the main reasons for the variability in the time of menopause onset [45].

There are many genetic and non-genetic determinants that affect the reproductive aging rate and the onset of menopause. It has been found that birth weight, presence of twins, mother's bad habits during pregnancy, as well as life history of smoking and exposure to hazardous substances (e.g., bisphenol A) are associated with an earlier onset of menopause in women [46–50].

The reproductive lifespan of a woman, which is controlled by ovarian function, also determines reproductive behavior and reproductive events during life. A large multicenter study has shown that early menarche and childlessness aggravate the risk of premature and early-onset menopause by 80 % and 2 times, respectively, while their combination can turn the risk of these conditions five times higher [51]. A recent systematic review and meta-analysis have found that age at menarche of ≥13 years and the fact of childbirth are associated with the later onset of menopause [52]. In another study, by contrast, early age at menarche (<11 years) and nulliparity have been shown to be risk factors for premature and early-onset menopause [51]. A population-based cohort study conducted on more than 100 thousand women has shown that women with a history of one or more childbirths as well as those with long history of breastfeeding (more than 6 months) have significantly reduced risks of premature and early-onset menopause compared to the nulliparous women [53]. It is expected that pregnancy and breastfeeding can, by suppressing ovulation, preserve ovarian reserve over the period they occur. It has been shown that a later age at the first full-term pregnancy as well as longer intervals between menarche and the establishment of a regular menstrual cycle are associated with a later age at menopause [54]. To clarify these associations, it is clearly necessary to continue research, in particular, the search for biomarkers of reproductive aging. With all the above factors taken into account, this will help determine the functional status of the reproductive system to subsequently predict age at menopause, which, as we already know, will significantly change women's lives.

## In Search of biomarkers of reproductive aging

### What are we trying to measure?

Up to the present day, researchers who are actively engaged in the search for biomarkers of aging have faced significant difficulties, since it is effectively impossible to measure all molecular and cellular processes that occur in the whole body with age due to versatility and multifactorial nature of this phenomenon as well as differences in aging rates of different tissues [55]. The problem could have been simpler for single organs. But, first, the reproductive system is not ovaries alone: it is a phylogenetic multi-level axis where other organs, including central nervous system structures, are structural units. And secondly, the ovaries have a distinctive feature—they contain both somatic and sex cells. The latter are defined as the “immortal” or “ageless” cells [56]. The divergence of the aging trajectory of somatic and sex cells is an important aspect of issues related to aging we should pay special attention to.

Aging of the body, as many gerontologists believe, is a consequence of accumulation of various damages in the cells (in particular, DNA damage) [57]. Female sex cells, like some somatic cells, cannot divide or live long and are expected to accumulate DNA damage which contributes to aging. However, they ensure the genetic information is transmitted from generation to generation, and the “old” gametes do not produce children that would inherit these age-related changes. In this context, the sex cells seem to avoid aging, contrasting themselves with the somatic ones.

For at least the past 100 years have the scientists been trying to explain this phenomenon [56]. According to one of the main theories of “immortality” of the germline, sex cells in the process of their development “get rid” of accumulated DNA damage through recombination repair, while the restoration of the native DNA structure is the main prerequisite for them to remain as effective as possible in terms of ensuring the birth of healthy offspring.

Another explanation for the “immortality” of oocytes may be the asymmetric cell division during maturation (after the first and second divisions of meiosis) resulting in a fully-formed egg cell and polar bodies being formed (which are subsequently reduced) [58]. This process is crucial for fertilization and subsequent development of the embryo; it allows the egg cell to “discharge” the accumulated molecular damage, including DNA damage, into one of their daughter cells (polar bodies). There is an opinion that such an asymmetric strategy has developed phylogenetically to preserve the population [58, 59].

However, the quality of egg cells still deteriorates with age, the mutational load on the offspring grows, and the risk of children with developmental abnormalities increases [60]. That is, the sex cells still show signs of aging at a certain point in time. This does not necessarily mean that the sex cells age in the same way as the somatic cells do. Perhaps it is just that the patterns of aging that occur in the somatic cells cannot be observed in sex cells. Yet, this fact is the main argument for those scientists who postulate that sex cells do not have any special mechanisms for preserving and maintaining youth. In their opinion, it's all about the so-called “barriers” that prevent the “old” egg cells from production of abnormal offspring [61]. Such “barriers” include the short-term ability of egg cells to fertilize after ovulation, cessation of the embryo development in “old”, defective gametes at different stages, atresia of the follicles during their successive growth, etc.

Whatever the causes and despite this feature of the sex cells it was mentioned above that the reproductive system ages faster than other systems in the body. And to detect it at early stages, we need to realize what we are going to ultimately measure (in terms of biomarkers). Despite the fact that, to some extent, all the structures of the reproductive axis age, according to the accepted dogma, reproductive aging is nowadays is defined as a decline in ovarian follicle number and decrease in the quality of egg cells in the ovaries [25].

The exact molecular mechanisms that determine the decline in oocyte/follicle quality are still to be determined. Having received evidence that the results of ART programs, as well as the ovarian follicle number, tend to deteriorate in correlation with age. The scientists have accepted the follicle number as an indirect marker of follicle quality [62, 63]. Although the true quality of egg cells can only be evaluated by the reproductive outcomes [64]. Some of the latest studies question applicability of the “quantity=quality” statement to young women, which may redirect the vector of research in reproductive medicine [65].

At the moment, there is still no international consensus on whether neo-oogenesis can be observed in the ovary or whether the pool of follicles is fixed and cannot increase or renew [66–69]. Some scientists do not rule out the possibility that stem cells give rise to new oocytes throughout a woman's life, but this process makes the least contribution to the overall pool of follicles and loses its relevance with age, so it alone is not sufficient to prevent the onset of menopause [70]. Ovarian aging processes would be associated with depletion of the available follicular reserve and/or with reduced ability to differentiate oogonial stem cells due to a decrease in the quality of their micro- and macroenvironment, which is associated with age-related changes in the hypothalamic-pituitary-ovarian axis and the entire organism [71]. Anyway, the signs of ovarian aging in clinical practice will (primarily) include decreased ovarian reserve, the concept most often used in relation to the ovarian follicular reserve.

Though widely spread, the concept of “ovarian reserve” causes confusion among scientists and clinicians when it comes to defining it. Commonly, the problem lies in attempts to evaluate the ovarian response to ovulation stimulation instead of true ovarian reserve, although these parameters are related [72]. This is probably why there is still no consistent approach to evaluating ovarian reserve. Some scientists in their attempts to resolve the confusion suggest using different terms for these concepts: “ovarian reserve” to define a pool of resting follicles, and “ovulatory potential” to define a pool of growing follicles (also referred to as the “functional ovarian reserve”) [73], although this approach is not common today.

There are also some difficulties in distinguishing the concepts of “decreased ovarian reserve” (DOR) and “premature ovarian insufficiency” (POI). DOR is not the equivalent of POI but rather applies to women with existing infertility and an increased risk of poor response to ovulation stimulation (poor ovarian response, POR) [74]. It is important to correctly interpret the diagnosed DOR, because despite the acknowledged trend of this condition towards an increase in incidence, the overdiagnosis rate is still high [75]. Clinical manifestations of subfertility as well as values of FSH >10 mIU/mL or AMH <1.0 ng/mL can serve as the criteria for diagnosing DOR [76]. The nature of DOR can be physiological when a woman is over 40 years old or pathological when she is younger, but the diagnosis is not associated with high FSH values typical for menopause or suppressed menstruation [77]. POI can be diagnosed in women under 40 if they develop oligomenorrhea/amenorrhea for at least 4 months with FSH levels of > 25 IU/l in [78].

Therefore, there are many definitions of the physiological and deteriorated (to some extent) ovarian function. But what are we really trying to measure? Since true ovarian reserve, including resting and growing follicles, can only be confirmed histologically, the currently known markers are more applicable to functional ovarian reserve — i.e., to growing follicles. To some extent, we can extrapolate these data to the indicator of true ovarian reserve, given that the number of growing and the number of resting follicles are intrinsically related to each other [79]. Leading scientific communities in the field of obstetrics and gynecology, including the American College of Obstetricians and Gynecologists (ACOG), postulate that the main purpose of ovarian function testing is to identify women with decreased ovarian reserve (DOR) and subsequently change the counseling tactics applied to such women both within ART programs and general gynecological and other practice.

It is known that any screening test has a number of parameters with sensitivity and specificity having special statuses. In the case of ovarian reserve markers (as the equivalent of reproductive aging), it is impossible to balance the sensitivity/specificity ratio without having either of the parameters decreased [80]; for these indicators, the requirements for their predictive value come to the fore. There is not much use in a marker that serves only to confirm a certain disease or condition in an individual or otherwise especially in reproductive medicine.

In this review, we will focus on the biomarkers of ovarian aging that are recommended due to their proven benefits and value of information they give. We would also like to focus on new promising areas of research in the field of reproductive aging as well as on the search for molecules that identify this process.

## Generally acknowledged markers of reproductive aging today

For a long time, the chronological age of female patients, nature of their menstrual cycle, certain hormonal parameters (such as estradiol (E2), follicle-stimulating hormone (FSH), inhibin B, etc.), ultrasound characteristics (number of antral follicles or total ovarian volume), and dynamic tests, such as the clomiphene citrate challenge test (CCCT), have been used as potential markers of ovarian reserve as well as for prediction of the reproductive potential in IVF programs and other areas [81]. According to the Practice Committee of the American Society for Reproductive Medicine, there is currently no single marker that should be recommended for evaluation of ovarian reserve; nor is there sufficient evidence that any combination of different tests improves the effectiveness of diagnosis [80]. Scientists also emphasize that the number of false-positive results of decreased ovarian reserve at diagnosis increases in the group of low-risk patients. In general, the committee's opinion on the most promising markers of ovarian reserve in terms of their prognostic value is consistent with the opinions of scientists from different countries and has been confirmed by numerous studies. With some limitations, they are still useful in practice and help clinicians make tactical treatment decisions.

Follicle stimulating hormone (FSH) is a biochemical marker and is a dimeric glycopeptide produced by the anterior pituitary gland. It has a regulatory effect on growth of follicles at the hormone-dependent stage of folliculogenesis. Clinical interpretation of actual (absolute) FSH levels is usually performed on the third day of the menstrual cycle, since its increase is better registered at the beginning of the first phase against a decrease in inhibin B and estradiol levels [82].

Individual variations in FSH levels have been observed during the menstrual cycle as well as from cycle to cycle in the same woman [83]. Studies have shown that the level of this hormone is relatively stable until the age of 33 and fluctuates slightly until the age of 40, but henceforth its fluctuation rate is constantly increasing, and as the ovaries age, these changes become significant [84]. In other words, the most pronounced increases in FSH levels are observed during periods when there already are changes in the duration and nature of the menstrual cycle that are characteristic of perimenopause. In such a case, there is an undoubted correlation with reproductive aging, although with its later stages thereof. Therefore, FSH cannot be considered a direct indicator of ovarian reserve [85] despite the simplicity and relatively low cost of the test. The FSH level has high specificity but low sensitivity and acts as a marker of poor ovarian response to ovulation stimulation [80]. Another limitation comes from the need to determine FSH and estradiol levels together, since, in women with decreased ovarian reserve, truly high FSH levels can seem conditionally normal when measured alone. This is due to the increased effect produced by FSH on growth of the remaining follicles at the beginning of the menstrual cycle and accordingly on the increase in the estradiol level. According to the principle of negative feedback, the latter leads to a decrease in the gonadotropin level [86].

Another biochemical parameter, which is currently the most promising and frequently used marker of ovarian reserve, is anti-Müllerian hormone (AMH). It is a glycoprotein that belongs to the transforming growth factor beta (TGF-β) family and is exclusively produced by granulosa cells of preantral and small antral follicles with a diameter of about 4 mm. In larger-diameter (6–8 mm) follicles, AMH is no longer secreted. Against a background of sharp drop in the AMH level, there occurs an increase in the activity of the aromatase enzyme and increased synthesis of estradiol, which indicates the presence of a feedback mechanism between the synthesis of estradiol by the granulosa cells of the dominant follicle and AMH. This hormone mainly exerts two types of effect on the ovary: it suppresses the primary stages of follicle growth and suppresses hormone-dependent growth and selection of preantral and small antral follicles [87]. However, it is only to be found out whether AMH exerts an extraovarian effect beside the intraovarian effect.

AMH shows less variability within and between the cycles compared to FSH [88]. For a long time, it has been believed that AMH can be determined at any phase of the cycle, but the conclusions of various researchers on the constancy of AMH levels are still controversial. Moreover, there is a reasonable opinion that AMH levels should be measured at the follicular phase of the menstrual cycle [89, 90].

AMH, along with antral follicle count (AFC), shows consistency with the pattern of oocyte loss seen in histology specimens [79], which allows us to consider it as a promising non-invasive biochemical marker of ovarian reserve. While AFC indicates the number of visible antral follicles with a diameter of 2–10 mm and is only indirectly related to true ovarian reserve (not only and not so much functional ovarian reserve), AMH is an indirect indicator of follicles that will be ready to start growing within the next 3–5 months [91]. Since the above directly depends on the number of primary follicles, this again points to the important role of AMH as the most accurate biomarker of ovarian reserve. Probably, the main limitation for AMH is the lack of a universally standardized method for determining this hormone.

AFC is one of the most popular methods of interpreting the functional state of the ovaries employed in current clinical practice. AFC, as well as AMH, varies less between menstrual cycles compared to FSH or, for example, ovarian volume. But the imaging examination methods are subjective [92]. AFC is considered the best marker of ovarian response to stimulation in IVF cycles. At the same time, the decrease in the antral follicle count clearly correlates with age, which allows us to consider it a marker of ovarian reserve when studying reproductive aging [72]. Currently, this is one of the most affordable and easy-to-perform diagnostic methods.

Although there has always been vigorous discussion on the ways to compare the effectiveness of the two key markers, AFC and AMH, with regard to different parameters of ovarian function [79, 93, 94], scientists agree on the high meaningfulness and prognostic value of these indicators and also highlight their advantages and disadvantages relative to each other [81]. In the case of such assessments, a number of factors that can affect the final result should be taken into account, including higher AMH blood levels being observed when ultrasound shows many small-diameter follicles as well as the inability to differentiate “healthy” follicles with granulosa cells capable of synthesizing AMH from follicles of the same size that have undergone atresia [63].

Other candidates for the role of biomarkers of ovarian reserve (reproductive aging) are less meaningful. According to the Practice Committee of the American Society for Reproductive Medicine, neither the basal inhibin-B level nor the ovarian volume or estradiol level values should be used as independent screening test indicators in the diagnosis of decreased ovarian reserve [80].

## Genetic markers of reproductive aging

Both menarche and menopause are fundamental events in a woman's life. The interval between the beginning of the reproductive life and its end is referred to as the reproductive lifespan, while a specific period within this interval is referred to as the reproductive age. While, with the knowledge of the female patient's reproductive age (not chronological or even biological), the doctor would only be able to assess her ability to conceive and give birth to a child at a specific time. On the other hand, information about the reproductive lifespan would offer more counseling options. Genetic markers may be the central point in this context, and, in this case, the strong genetic component of age at menarche and menopause promises scientists good opportunities and prospects for research [95].

Currently, the only genetic marker widely used in clinical examination of patients with suspected POI is determination of 5'-UTR a triplet-base-repeat (CGG, (Cytosine Guanine Guanine) in the FMR1 gene (fragile X mental retardation 1) localized on the long arm of the X chromosome at locus Хg27.3. This gene is considered mainly responsible for one form of mental retardation (X-linked mental retardation) when the number of triplet repeats are located at the 5' untranslated region of the gene exceeds 200 (complete mutation). The expression of the product of this gene also plays an important role in formation of physiological ovarian reserve. The increase in triplet CGG repeats in the FMR1 gene within the range of 55–200 (gene premutation) is well known to reproductologists, since it is this range of CGG repeats that is most often associated with POI and infertility. Another deviation is represented by the number of repetitions being within the range of 35–54 (the “gray zone”), which is typical both of patients with the conventional form of POI and patients with periodically fluctuating FSH levels [96, 97]. However, published findings in this field are very contradictory. Some scientists do not associate such a deviation with a specific ovarian-insufficiency-prone phenotype [98]. It should be noted that mutations in the FMR1 gene are also associated with decreased ovarian reserve (DOR) [99]. Thus, we can conclude that deviations in the normal number of Cytosine-Guanine-Guanine repeats in the FMR1 gene somehow indicate an increased likelihood of developing POI and early-onset menopause in the future, which means that such patients require careful annual monitoring of the state of ovarian reserve and general health.

Recent studies have provided a large amount of previously unknown information about the molecular and genetic basis of reproductive aging [100]. The search for genes that are in one way or another involved in the processes reducing ovarian reserve (physiological or pathological) was carried out using the candidate gene approach involving selection of genes based on understanding of the pathophysiology of reproductive aging. This way, fertility control genes were identified and the relationship between monogenic mutations (mutation of the follicle-stimulating hormone receptor (FSHR) gene), mutation of the luteinizing hormone receptor (LHR) gene), or the inhibin A gene (INHA), and premature ovarian insufficiency was revealed [101]. Another example is the signaling pathways determined according to the same principles and ensuring the modulating effect produced by growth factor proteins (in particular, transforming growth factor beta (TGF-β): theca-derived BMP4 (bone morphogenetic protein 4) and BMP7, granulosa-derived BMP6, oocyte-derived growth differentiation factor 9 (GDF9), as well as BMP5 and BMP15) on intraovarian physiological interrelations between oocytes, granulosa cells, and theca [102], which also contribute to the etiology of POI.

A kind of revolution was made just over 10 years ago by genome-wide association studies (GWAS), the philosophy of which is to identify the interrelations between disease and genetic variants (polymorphisms) of individual nucleotides [103]. Despite forecasts that this type of research would be an intermediate technology in understanding the genetics of various medical conditions and might soon be replaced by more advanced methods, we still receive information about previously unknown biological pathways associated with reproductive aging [104, 105]. For example, a recent meta-analysis of data from 53 GWAS involving a total of 70,000 female subjects has identified 44 loci associated with age at natural menopause, 2/3 of which are associated with DNA-damage reparative response (including genes Helicase, POLQ Like (HELQ), crucial member of the ATM-mediated DNA double-stand breaks repair family of genes (BRCA1), minichromosome maintenance complex component 8 (MCM8), MutS Homolog 5 (MSH5), Exonuclease 1 (EXO1), DNA Polymerase Gamma, Catalytic Subunit gene (POLG), and others) [106]. The most significant results on genetic polymorphisms associated with premature ovarian insufficiency have been obtained with regard to the loci where the DNA repair genes are located (Stromal antigen 3 (STAG3), Synaptonemal complex central element 1 (SYCE1), Scaffold Protein Involved In DNA Repair (SPIDR), PSMC3 Interacting Protein (PSMC3IP), ATP-dependent DNA helicase homolog (HFM1), MutS Homolog 4 (MSH4), MSH5, minichromosome maintenance complex component 8 (MCM8), MCM9, Nucleoporin 107 (NUP107), signaling pathways of mRNA transcription, and translation (Eukaryotic Translation Initiation Factor 4E Nuclear Import Factor 1 (eIF4ENIF1), KH RNA Binding Domain Containing, Signal Transduction Associated 1 (KHDRBS), and already known candidate genes of POI (Spermatogenesis and oogenesis helix-loop-helix 1 (SOHLH1), Follicle Stimulating Hormone Receptor (FSHR) [104, 107].

DNA repair appears to play a significant role (much more important than previously thought) in the decline in the ovarian follicle number. Oocytes can be highly susceptible to DNA damage occurring in response to metabolic and environmental impacts, including due to prolonged cell cycle arrest [106]. DNA damage repair is also an important component of meiosis and mitotic division of oogonia [108]. Ultimately, the instability of the genome caused by the inability to repair DNA breaks disrupts the vital activity of oocytes, contributing to death thereof.

A series of GWAS studies suggested that certain items in the sets of genes responsible for age at natural menopause and POI are the same, which revealed common pathways of genetic susceptibility of these two conditions [109]. Other equally important findings from these studies are data on GWAS loci associated with age at menopause and located in or near the genes responsible for controlling the hypothalamic-pituitary-ovarian axis (such as Fibroblast Growth Factor Receptor 1 (FGFR1), SRY-Box Transcription Factor 10 (SOX10), Chromodomain-Helicase-DNA-binding protein 7 (CHD7), Gonadotropin Releasing Hormone 1 (GNRH1), KiSS-1 Metastasis Suppressor (KISS1), Tachykinin Precursor 3 (TAC3), as well as KISS1 Receptor (KISSR), Tachykinin Receptor 3 (TACR3) and Gonadotropin Releasing Hormone Receptor (GNRHR), which indicates that the beginning and end of the reproductive lifespan are highly influenced by the central regulation [27, 106].

Allelic heterogeneity is also observed in the loci of genes associated with the age at menopause, energy homeostasis, regulation of lipid metabolism, etc. These findings largely shed light on the gaps in understanding of the interrelation between reproductive aging and various adverse health outcomes, which have been confirmed by numerous epidemiological studies [110].

Physiological and pathological reproductive phenotypes have polygenic nature [111]. Diagnosing polygenic traits, in particular, those of reproductive aging, is difficult due to the complexity of determination of the effects produced by specific genes and their contribution to formation of phenotypic traits. When applied to the above, the methods used in GWAS have their limitations. Such limitations are associated with identification of common variants, which are widely represented in the genome and the impact of which on the development of the disease is insignificant [112]. This molecular paradigm, along with additional limitations, has pushed scientists to develop new approaches to genetic research proceeding from common variants with little effect to highly penetrant rarer variants — Next-Generation Sequencing (NGS).

This approach is promising for the molecular diagnostics of reproductive aging (which is a complex heterogeneous phenomenon) and, in particular, diagnostics of premature ovarian insufficiency. Despite the established contribution of the genetic component to the pathogenesis of POI (including for the isolated form) and identification of several dozen genes involved in this condition, there is no complete understanding of the genetics of POI, and most patients do not receive genetic diagnoses [111]. In ongoing studies, NGS has already demonstrated its extensive ability to shed light on the mysteries of the genetic component of POI [113, 114]. Despite the limitations of a relatively small sample size or incomplete panel of genes associated with this condition, new causal variants have been identified in genes that, without NGS, would hardly be considered as priority candidate genes. In particular, variants of genes encoding important factors involved in DNA damage response (MCM9, FA Complementation Group A (FANCA), FA Complementation Group L (FANCL) etc.) [115–117], variants of genes involved in key biological processes such as meiosis and follicle development (Spermatogenesis and oogenesis helix-loop-helix 1 и 2 (SOHLH1, SOHLH2), STAG3, HFM1, MSH4, MSH5, Synaptonemal complex protein 2-like (SYCP2L), and a number of others) [117, 118], or heterozygous variants in Eukaryotic Translation Initiation Factor 2B Subunit Beta (EIF2B2) encoding the translation initiation mechanism [119].

Overall, GWAS and NGS have identified common genetic and molecular pathways in reproductive aging. Some of the results obtained have already been confirmed in a number of other studies, and some have yet to be clarified. Another important conclusion made after more than 10 years of successful use of such large-scale studies is that the identified genes are often difficult to associate with the development of medical conditions since most of them do not encode proteins and many features can be considered quantitative due to the subtle interaction of many genes. Another thing to keep in mind is that in the case of polygenic inheritance we are dealing with the phenomena of incomplete gene penetration and varying relevance to the disease progression, which makes it hard to identify responsible candidate genes. Scientists suggest that the candidate genes for the disease progression function consolidated in networks, and it is the discovery of these networks and understanding of the principle of their action that is a priority for future research. In this regard, there is great potential in using individual approaches to each case of POI, NGS, and sequencing with pre-analyzed coding and regulatory regions of genes for diagnostic and prognostic purposes.

## Mitochondrial dysfunction as a marker of reproductive aging

The etiology of early and premature menopause, which is an example of premature reproductive aging, is extremely diverse. Chronic conditions that maintain inflammation in the body can indirectly or directly cause premature reproductive aging. Premature ovarian insufficiency is associated with genetic predisposition, autoimmune diseases, and infectious diseases [120, 121]. This condition can be a symptom of monogenic, polygenic, and chromosomal diseases as well as that of a number of syndromes [122]. However, the etiology remains unknown in most cases, and idiopathic cases prevail in the incidence structure of POI.

The mechanisms underlying reproductive aging are complex and their pathological signaling pathways, apparently, run into numerous intersections as the disease progresses. There is evidence that premature and early-onset menopause are associated with mitochondrial dysfunction [123, 124]. The underlying causes and pathways of mitochondrial dysfunction are extremely significant to identify, as this may later lead to the development of anti-aging agents and facilitate obtaining new data on potential markers of reproductive aging.

As is known, being widely recognized in the scientific community, mitochondrial free radical theory of aging puts Increased production of mitochondrial reactive oxygen species (ROS) and oxidative stress at the center of processes that contribute to aging of cells [125, 126]. Mitochondria are energy-generating systems of the body that control free radical levels and generate ATF, thus being the most important regulators of cellular survival or death [127, 128]. Also, mitochondria are the only cell organelles that have their own, mitochondrial DNA (mDNA) [129].

The number of mitochondria and mtDNA copies varies in different cell types depending on their energy needs. Oocytes, the largest cells in the body, require the maximum number of mitochondria and contain the largest amount of mtDNA [130]. They require energy to spend on maintaining normal transcription and translation processes during their maturation as well as on fulfilling the increased energy needs during the post-fertilization stage of embryonic development [131].

Studies demonstrate that decreased mitochondrial biogenesis, impaired mitochondrial homeostasis, and free radical imbalance, associated with the latter, play a critical role in ovarian aging [132]. Qualitative and quantitative changes in mtDNA are central factors in this process. High content of free radicals can incite oxidative damage, chain breaking, and mtDNA mutations [131]. Other mechanisms of mitochondrial dysfunction include reduction of the transmembrane mitochondrial potential and disruption of the electron transport chain (ETC) function [132]. These processes can initiate apoptosis in the cell, which, in the case of oocytes, leads to depletion of the follicular reserve. Animal experiments have clearly demonstrated the association of mtDNA mutations with decreased ovarian reserve and decreased fertility in mice [133]. Female carriers of mtDNA mutations in ART programs have also shown decreased ovarian reserve [134].

The number of mtDNA is significantly reduced in the oocytes of old animals compared to young ones [123]. This also correlates with the results of fertilization in ART programs [135]. Finally, a reduced number of mtDNA is typical of oocytes in patients with ovarian insufficiency [136].

Scientists believe that changes in mtDNA act as markers of mitochondrial aging, while mitochondrial aging reflects the essence of systemic aging [137]. Mitochondrial function indicators may act as markers of biological aging, including reproductive aging. The mitochondrial theory of aging and the role of oxidative stress can be supported by the pronounced therapeutic effect of mitochondrial-targeted antioxidants produced on oocytes [138]. In the context of reproductive technologies, the aging reproductive process is seen as poor response to ovulation stimulation in ART programs as well as poor quality of the resulting embryos. The researchers found that not only do low levels of mtDNA correlate with POI, but they are directly proportional to the stage of reproductive aging; the lowest levels of mtDNA have been observed in patients with complete premature ovarian insufficiency, higher levels have been recorded in patients with poor response to ovulation stimulation, and the highest levels of mtDNA have been observed in patients with normal ovarian function [139]. Interestingly, the reduced number of mtDNA copies in the POI arm and poor ovarian response were significantly lower than those in the control arms (women with normal ovarian reserve and older women with natural menopause). The apparent downward trend of mtDNA levels between healthy women with preserved ovarian reserve and women with expanded clinical presentation of POI suggests that the number of mtDNA may be a potent marker of reproductive aging. Another potential application area is ART programs, where the number of mtDNA may act as a predictive marker for embryo quality and viability [135].

Mutations of mtDNA accumulate over time and worsens cellular function. Based on the above, the accumulation of mtDNA mutations is now considered as the “biological clock”, which is of great interest today [131, 140]. Also, the metabolic function of mitochondria can be determined with the use of skeletal muscle biopsy (invasive procedure) and phosphorus magnetic resonance spectroscopy [141, 142]. Both methods have their limitations, and, at the moment, the indicators of mitochondrial function need to be standardized for clinical application. It has also recently been shown that the use of technological solutions that detect discrete changes in cellular bioenergetics in real time, such as Seahorse (Agilent Technologies), could be useful for assessing the quality of oocytes and early embryos [143]. Specialists in reproductive medicine can benefit from these markers, since both determination of dysfunctional oocytes in ART programs and determination of the biological age are extremely important when consulting the patients and determining management tactics.

## “Aging clocks” as a potential biomarker of reproductive health

Chronological age is clearly a determining factor of aging and accumulation of aging-associated diseases. However, among the elderly, the rate of these processes and outcomes is significantly heterogenous. Determination of biological age as a true indicator of aging has become a priority for gerontology recently, and the scientific research in the field of identification and implementation of the corresponding biomarkers continues [144]. In the future, they will be able to help develop approaches and interventions to increase life expectancy and mitigate the effects of aging in humans [145].

Cells “report” aging by stopping the cell cycle and triggering the so-called secretory aging-associated phenotype (“senescence-associated secretory phenotype” (SASP). This phenotype is associated with the expression of typical biomarkers (proinflammatory cytokines, chemokines, growth factors, etc.) [146]. Attempts are being made to “measure” the biological age based on the quantitative assessment of aging cells in the body by identifying these markers in the blood [147]. These markers are not specific, and the most promising method currently is determination of these molecules in biopsy samples of different tissues, e.g., in ovarian tissues in patients with premature and early-onset menopause.

In recent years, significant progress has been made in the development of the so-called epigenetic “aging clock”. Epigenetic mechanisms control gene expression programs, thereby determining the cellular homeostasis. The main epigenetic mechanisms come down to DNA methylation and processes that induce changes in the state of chromatin (associated with histone modification and other molecular events) and non-coding RNA [148]. DNA methylation is the process of modification of DNA molecules without changing the nucleotide sequence. This process governs a large number of genetic mechanisms in the cell (e.g., replication, transcription, DNA repair) and also is a mechanism for cellular and tissue differentiation as well as for repression and discrimination of genes [149]. During life, when exposed to environmental factors as well as during aging, an individual develops significant changes in the patterns of DNA methylation. In 2013, both Horvath [150] and Hannum et al. [151] published their works on the epigenetic aging clock that uses the state of methylation sites and involves a mathematical model calibrated by changes in DNA methylation during physiological aging to determine the biological age of a person. Later in 2015, scientists proposed using deep learning models to predict age with the help of simple biochemical test results and cell counts [152], transcription profiles [153], face images [154], and brain MRI [155]. One of the recent developments is represented by the aging clock based on taxonomic profiling of the gut microbiome composition [156]. Interestingly, the gut microbiome composition is associated with the level of estrogens, which opens a new area of research [157].

Nowadays, telomere length is considered to be one of the most meaningful markers of biological age [158]. Telomeres are repetitive (TTAGGG) n-sequences that cap the linear ends of chromosomes. Telomere length decreases with age. An insufficient number of TTAGGG repeats leads to aging and subsequent death of cells [159]. Accelerated telomere shortening is associated with a variety of medical conditions. In 2006, a telomeric theory of reproductive aging in females was proposed, according to which the age-related decrease in the quality of oocytes is the result of progressive shortening of telomere length [160]. The authors of the theory were able to test it by experimentally shortening the length of telomeres in mice, as a result of which the animals developed the female reproductive aging phenotype typical of humans (namely, deterioration of the mitotic and meiotic chromosomal divergence, reduced number of chiasmata, arrested embryonic development, etc.). Some studies show evidence of telomeric DNA deficiency occurring in aneuploid oocytes and embryos during IVF cycles [161]. Such studies are important for the progress of knowledge the biology of female aging. However, it is still not fully clear whether the shortening of telomere length is a cause or a consequence of aging [162].

The cause-effect relationship between the processes of reproductive and general somatic aging is complex. In 2016, two groups of researchers tried to identify the interrelations between biological aging, accumulation of diseases, epigenetic changes, and reproductive aging [17, 163]. The main interest was drawn to the question of how ovarian function can regulate general aging processes in females. The conclusions allowed the authors to construct several possible models of causes and consequences of these processes.

One model suggests that certain factors (genetics, environmental factors, social factors) independently predispose affected individuals to both accelerated biological and accelerated reproductive aging as well as to accumulation of various diseases. In this case, the true biological age, which differs from the chronological age, can be considered a marker of accelerated reproductive aging, while its determined value may be sort of an indicator of age at menopause.

According to another model, the loss of ovarian function (especially after /premature menopause resulting from ovariectomy) can be considered the main cause of acceleration of general aging processes, increase in epigenetic age, and accumulation of multimorbidity. In this case, determination of biological age provides important additional information about the patient's state of health, which may affect therapeutic strategies and recommendations (including those regarding the ability to bear a child).

As is known, different organs age at different rates, moreover, biomarkers appear to track different biological processes and, therefore, could have low correlation with each other. Obviously, sets of biomarkers, rather than any single biomarker, are the most effective means of assessing the health status; in the case of aging biomarkers, scientists suggest using the most holistic approach to collecting information about aging structures of the body for comprehensive assessment of the obtained biomarkers [164]. Development of bioinformatics and artificial intelligent, their potential for processing of omics big data, and training of age prediction models using data from specific populations are the current goals in this area of research.

## CONCLUSIONS

**Figure 2:**
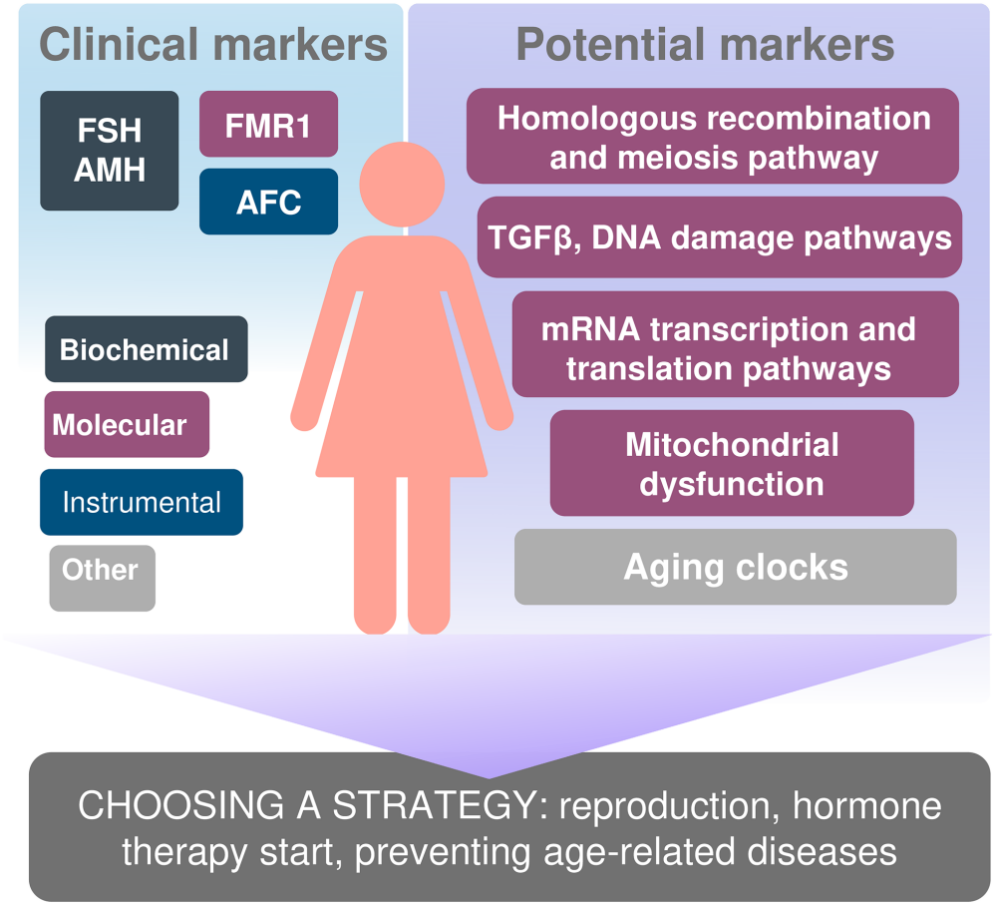
Biomarkers of reproductive and somatic aging ana personification the approach to managing patients.

With the modern tendency of women emancipation, the need to make them aware both of their reproductive potential and the reproductive lifespan, which is important for their social and labor functioning, is growing. Needless to mention, we would like the markers of these parameters to be highly meaningful, low-cost, and able to predict age at menopause and the endpoint of the reproductive period as early as possible. If a decrease in ovarian reserve is diagnosed at a younger age, we can personalize the approach to managing patients and take possible effective measures aimed at facilitating their reproductive function capacity (Figure 2).

In the modern world, there is a certain illusory opinion that delayed childbearing is not a problem in the context of the developing modern technologies, including IVF programs. However, it is not always possible to solve the problem of infertility, even with the use of the latest technologies. Perhaps, availability of prognostic information about the true reproductive lifespan would allow women to correctly and timely set the necessary priorities in life. Determination of the true biological age of a woman may be an additional and very important option, regardless of whether we consider it as a predictive marker of reproductive health or as an indicator of overall health and a powerful tool for selecting the most suitable approaches to treatment and prevention of various diseases.

The number of potential biomarkers of reproductive aging continues to grow, and it is necessary to carefully plan the strategies of their development. To date, prediction of the length of a woman's reproductive lifespan is still a goal that we have not achieved yet. Future studies should be aimed at identification of more markers, validation thereof, and widespread implementation in clinical practice.
